# Predicting the quality of ryegrass using hyperspectral imaging

**DOI:** 10.1186/s13007-019-0448-2

**Published:** 2019-06-06

**Authors:** Paul R. Shorten, Shane R. Leath, Jana Schmidt, Kioumars Ghamkhar

**Affiliations:** 1 AgResearch, Ruakura Research Centre, Private Bag 3123, Hamilton, 3240 New Zealand; 2 AgResearch, Grasslands Research Centre, Private Bag 11008, Palmerston North, 4442 New Zealand

**Keywords:** Hyperspectral imaging, NIRS, Forage, Ryegrass, Pasture, Sugar, Nitrogen

## Abstract

**Background:**

The quality of forage plants is a crucial component of animal performance and a limiting factor in pasture based production systems. Key forage attributes that may require improvement include the sugar, lipid, protein and energy contents of the vegetative parts of these plants. The aim of this study was to evaluate the potential capacity of hyperspectral imaging (HSI) for non-invasive assessment of forage chemical composition. Hyperspectral image data within the visible near-infrared range into the extended near-infrared covering 550–1700 nm wavelengths were obtained from 185 accessions of ryegrass (*Lolium perenne*), which were also analysed for 13 forage quality attributes.

**Results:**

Medium to high predictive power was observed for the HSI models of total sugars (R^2^ validation of 0.58), high molecular weight sugars (R^2^ validation of 0.63), %Ash (R^2^ validation of 0.50) and %Nitrogen (R^2^ validation of 0.70). Significant HSI models with low R^2^ validation of 0.1–0.5 were also obtained for low molecular weight sugars, NDF (%), ADF (%), DOMD (% DM), ME (MJ/kg DM), DM (%), Ca (mg/g) and OM (%). We also observed significant differences in the chemical composition between the pseudostems and leaves of the plants for each accession. The power of HSI for prediction of these differences within plants was also demonstrated.

**Conclusion:**

This study paves the way for the HSI technology to be used for in-field estimation of forage composition attributes in perennial ryegrass. This will allow more rapid genetic-based selection and breeding for a trait that is normally expensive to measure providing a cheaper, non-destructive and high throughput screening tool.

## Background

Genomic selection can be effectively used to increase forage and animal productivity by selecting traits of interest in forages [1]. Among these traits, forage composition is one of the hardest and most costly traits to measure as it requires harvest and manual separation. Another limitation to the measurement of this trait is that the standard method of measuring forage composition requires collection and destruction of the plant material. Therefore, non-invasive technologies from which models can be developed to predict the composition of forage would be highly valuable [2]. A body of research has demonstrated the potential of non-invasive near-infrared spectroscopy (NIRS) based methods (dried and ground samples) to estimate the components of forage [3–6]. Other non-invasive technologies including Hyperspectral Imaging (HSI) systems [7–10] have been used to develop predictive models for forage quality and quantity. If accurate prediction models can be developed, such technologies will offer the potential for faster and higher throughput prediction of forage quality attributes and hence streamlining more rapid genomic selection for quality traits.

Near-infrared spectroscopy (NIRS) is based on utilizing the interaction of electromagnetic radiation with matter to detect the characteristic chemical signatures of target materials [2]. NIRS instruments based on single point measurement typically require a multitude of replicate scans of the target object for improved prediction of attributes. NIRS instruments can be used to predict the attributes of heterogeneous materials provided a sufficient number of replicate scans are obtained to account for this heterogeneity [11] although this adds extra time and cost to applications. NIRS has been used in perennial ryegrass to estimate leaf relative water content [12]. HSI instruments, however, are capable of capturing both spectral and spatial information [2]. These features make the technology suitable for automated, rapid and large-scale screening of forage. HSI systems have been used at plot, paddock, farm and catchment scales to determine type of forage as well as the quality of forage [7–10, 13, 14], although there is less information at the plant scale, which is the scale of interest for plant breeders. The deployment of the HSI technology, like any other technology, for forage analysis is dependent on the time and spatial scales of interest. Furthermore, because model calibrations are dependent on the spatial scale of interest as well as the lighting conditions, care must be taken when transferring calibration equations between spatial scales. This is specifically true when the forage is heterogeneous, and lighting and forage geometry only allow for observation of a subset of the sward in which case, a calibration transfer technique must be employed.

The targeted selection of new forages for optimal animal production and environmental outcomes in pastoral-based systems requires detailed information on forage composition. Information on the temporal (diurnal and seasonal) and spatial (between plants in the field as well as within the sward) variation in the composition of forage is also required to assess the performance of new forages and their interaction with the farm system. Forage quality traits of interest include the contents of total sugars, high molecular weight (HMW) sugars, low molecular weight (LMW) sugars, ash, calcium (Ca) and nitrogen [1, 15]. In addition, more complex and calculated composition traits such as neutral detergent fibre (NDF), acid detergent fibre (ADF), digestible organic matter in dry matter (DOMD), metabolisable energy (ME), and organic matter (OM) are the gold standard traits in forages [1, 15]. The ME content of forage plays the central role in animal growth, maintenance and production. It also provides a measure of production potential, although production is dependent on the other components of the forage as well [15]. NDF is an indication of the digestibility of cellulose, hemicellulose and lignin which comprise the cell wall components of the plant structure. ADF is a measure of the digestibility of the cellulose and lignin components only. The ash in forage consists of mineral elements such as sulphates, chlorides, and phosphates, which provide no energetic value to the animal. DOMD represents the organic matter in dry matter (DM), which is residual dry weight of the forage after the removal of moisture. The water soluble carbohydrate (WSC) includes mono- and di-saccharides, oligosaccharides and fructans (LMW and HMW sugars). Water soluble carbohydrates are the most digestible components of ryegrass and play an important role in the complex process of ruminant digestion [16]. Water soluble carbohydrates also provide a primary substrate for ruminal microbiota to breakdown of plant protein, which then becomes available for animal growth and maintenance. High WSC forage also potentially increases milk production of dairy cows while reducing the excretion of urinary nitrogen. The latter is a significant environmental problem in pastoral-based agricultural systems [16]. The concentration of the soluble sugars within ryegrass also exhibits a diurnal pattern that is seasonally dependent on the time course of photosynthesis [17–20]. Reducing the nitrogen content of forage also provides another strategy to reduce the nitrogen intake of the animal and therefore reduce urinary nitrogen levels and nitrogen losses from pasture [21–23].

The purpose of this study is to develop and test HSI models to predict the chemical composition of different genotypes of perennial ryegrass. Another goal is to examine the differences in the chemical composition of the leaves and pseudostems of the plants. The role of different wavelengths in the detection of different forage quality traits was investigated. Also, the likely predictive power of different wave ranges of very near-infrared (VNIR) and extended NIR was explored. An examination of the utility of a range of chemometric methods to predict the chemical composition of ryegrass forage was also conducted.

## Results

**Fig. 1:**
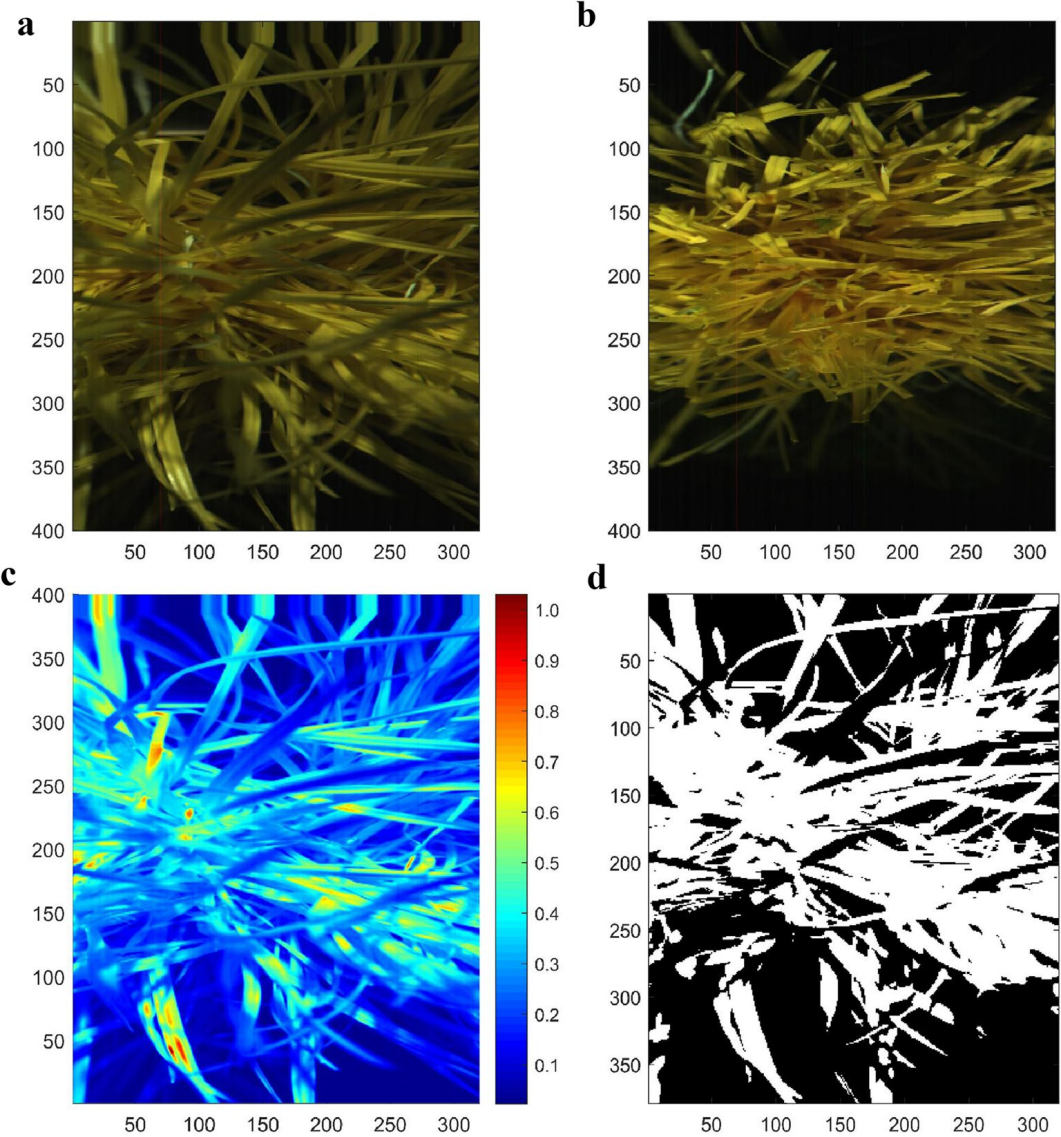
**a** Raw HSI image (320 pixels per line by 400 lines per image) of the ryegrass blades (at 558, 740 and 937 nm wavelengths). The image was captured directly above the plant. **b** Raw HSI image of forage of the ryegrass pseudostems after harvesting the top half of the plant (at 558, 740 and 937 nm wavelengths). The image was captured directly above the plant. **c** Forage heat map of ryegrass at 1080 nm (red denotes high reflectance and blue denotes low reflectance). **d** Region of interest (ROI) for the ryegrass (white pixels)

**Fig. 2:**
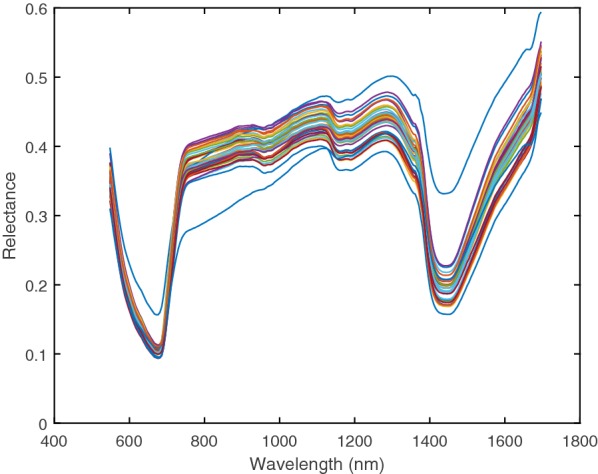
Distribution in mean forage reflectance spectra in the ROI for blades across the 37 ryegrass genotypes

Raw HSI images of forage blades and pseudostems are shown in Fig. 1a, b respectively. The forage heat map at 1080 nm is shown in Fig. 1c for the blades image in Fig. 1a. The region of interest (ROI) for the blades image in Fig. 1a is shown in Fig. 1d. The distribution in mean forage reflectance spectra in the ROI across all 37 genotypes is shown in Fig. 2. There is significant variability between genotypes in the mean forage reflectance spectra in the ROI for blades samples (*P* < 0.001). Reflectance minima are at 1000 nm, 1200 nm and 1450 nm and are consistent with water absorbance bands and forage reflectance spectra obtained from dried and milled samples [3].

**Fig. 3:**
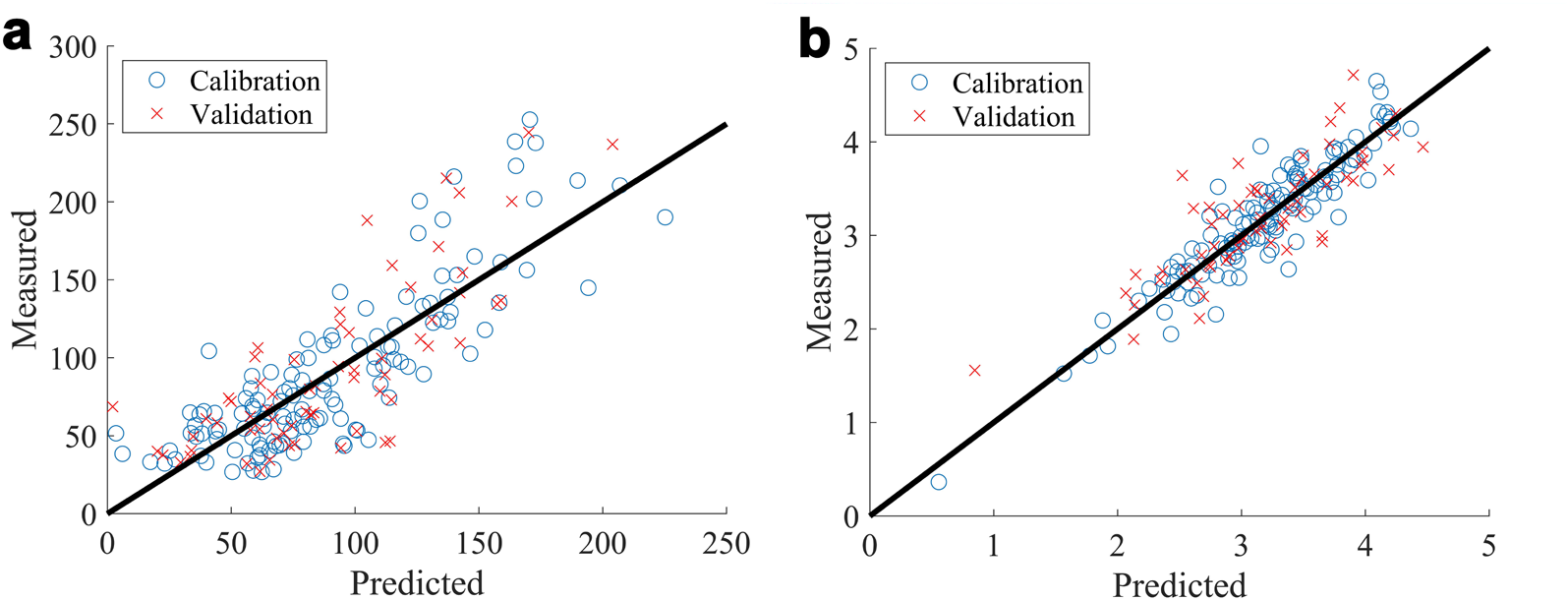
**a** Validated model performance for total sugars expressed as mg/g for the 2016 trial (BL + PS HSI reflectance spectra analysed by PLSR (SNV) model; Validation R^2^ = 0.58, n = 66, LV = 12, RMSE = 34.4 mg/g, intercept = 10.1 ± 9.6 mg/g, slope = 0.93 ± 0.10, bias = − 3.8 mg/g). **b** Validated model performance for Nitrogen % for the 2016 trial (BL + PS HSI mean reflectance spectra analyzed by PLSR (SNV) model; Validation R^2^ = 0.70, RMSE = 0.35%, n = 64)

Prediction models were calibrated and validated using the hyperspectral images and the wet chemistry data (partial least squares regression (PLSR) models). Comparisons of the best candidate calibration models for the BL + PS (Fig. 3) and BL datasets are described in Table 1) where model performance was similar for each dataset. The performance of the model for the prediction of total sugars in the BL + PS dataset is shown in Fig. 3a (R^2^ validation = 0.58, n = 66, LV = 12, RMSE = 34.4 mg/g). Average-high model performance was also observed for the prediction of HMW sugars for the BL + PS dataset (R^2^ validation = 0.63, n = 66, LV = 12, RMSE = 21.6 mg/g). The model performance for % Nitrogen for the BL + PS dataset is shown in Fig. 3b (R^2^ = 0.70, n = 64, LV = 20, RMSE = 0.35%). HSI models (R^2^ validation of 0.1–0.5) are also obtained for Ash (%), NDF (%), ADF (%), DOMD (% DM), ME (MJ/kg DM), DM (%), Ca (mg/g) and OM (%).

**Table 1:** Model performance for the 13 forage attributes based on full spectrum

Variable	HSI	# LV PLSR	Calibration							Validation						
			R^2^	RMSE	# images	Min.	Max.	Mean	SD	R^2^	RMSE	# images	Min.	Max.	Mean	SD
LMW (mg/g)	BL + PS	8	0.54	13.90	131	22.3	127.4	53.9	20.4	0.26	17.60	64	23.0	121.3	54.1	20.3
HMW (mg/g)	BL + PS	12	0.68	19.90	129	2.4	167.5	36.2	35.1	0.63	21.60	66	3.1	144.1	37.1	35.0
Sugars (mg/g)	BL + PS	12	0.70	28.90	129	26.7	252.8	90.0	52.2	0.58	34.40	66	27.1	244.5	91.5	52.8
Visual yield (%)	BL + PS	4	0.43	2.85	25	11.4	26.5	19.9	3.7	0.12	3.24	12	14.6	25.9	20.3	3.3
NDF (%)	BL + PS	11	0.38	2.53	123	38.7	53.1	45.9	3.2	0.19	2.98	62	38.7	53.8	45.9	3.3
ADF (%)	BL + PS	16	0.59	1.39	123	21.1	30.2	25.7	2.2	0.36	1.68	62	21.6	30.2	25.9	2.1
Nitrogen (%)	BL + PS	20	0.86	0.25	127	0.4	4.65	3.2	0.7	0.70	0.35	64	1.6	4.72	3.21	0.6
DOMD (% DM)	BL + PS	9	0.43	1.36	120	60.0	69.4	65.2	1.8	0.25	1.50	61	61.2	69.2	65.3	1.7
ME (MJ/kg DM)	BL + PS	9	0.43	0.22	120	9.6	11.1	10.4	0.3	0.25	0.24	61	9.8	11.1	10.4	0.3
DM (%)	BL + PS	13	0.45	0.52	124	92.9	96.4	94.5	0.7	0.13	0.64	63	93.0	96.2	94.5	0.7
Ash (%)	BL + PS	21	0.70	0.66	125	5.7	12.6	9.8	1.2	0.50	0.80	62	6.5	12.2	9.9	1.1
Ca (mg/g)	BL + PS	6	0.18	0.97	127	1.6	7.9	4.3	1.1	0.17	0.91	60	2.5	7.2	4.3	1.0
OM (%)	BL + PS	12	0.62	0.91	125	80.7	89.5	84.6	1.5	0.43	1.04	62	81.2	88.4	84.6	1.4
LMW (mg/g)	BL	8	0.51	13.60	121	22.7	127.4	51.9	19.3	0.26	17.40	59	23.1	121.3	52.5	20.0
HMW (mg/g)	BL	13	0.73	16.90	121	2.4	167.5	32.8	32.5	0.71	18.50	59	3.1	136.2	34.5	33.8
Sugars (mg/g)	BL	12	0.71	27.00	119	26.7	252.8	85.5	50.1	0.54	32.80	61	27.1	244.5	85.1	47.8
Visual yield (%)	BL	4	0.42	2.86	25	11.4	26.5	19.9	3.7	0.05	3.35	12	14.6	25.9	20.3	3.3
NDF (%)	BL	10	0.35	2.56	116	38.7	53.1	45.7	3.2	0.14	3.01	54	38.7	53.8	45.5	3.2
ADF (%)	BL	8	0.34	1.78	113	21.1	30.2	25.6	2.2	0.41	1.61	57	21.6	30.2	25.8	2.1
Nitrogen (%)	BL	9	0.61	0.34	115	1.9	4.6	3.3	0.5	0.52	0.39	61	2.1	4.7	3.3	0.6
DOMD (% DM)	BL	9	0.47	1.27	112	60.6	69.4	65.3	1.7	0.33	1.44	54	61.2	69.2	65.3	1.7
ME (MJ/kg DM)	BL	9	0.47	0.20	112	9.7	11.1	10.4	0.3	0.33	0.23	54	9.8	11.1	10.4	0.3
DM (%)	BL	9	0.33	0.58	113	92.9	96.4	94.5	0.7	0.11	0.63	59	93.0	96.2	94.5	0.7
Ash (%)	BL	21	0.87	0.43	116	5.7	12.6	9.9	1.2	0.55	0.77	56	6.5	12.2	9.9	1.1
Ca (mg/g)	BL	5	0.18	0.96	117	1.6	7.9	4.4	1.0	0.08	0.93	55	3.0	7.2	4.4	0.9
OM (%)	BL	15	0.72	0.80	113	80.7	89.5	84.6	1.5	0.50	0.98	59	81.2	88.4	84.6	1.4

Models are based on the mean reflectance HSI spectra analysed by PLSR model (Blades (BL) + pseudostems (PS) and blades (BL) only; Standard Normal Variate (SNV) preprocessing

We also examined the ability of a variety of multivariate methods to predict the forage attributes. Our comparison of the different methods for nitrogen prediction on the BL + PS dataset is summarized in Table 2. Partial least squares regression methods provided the best predictions on the validation dataset. Gaussian process regression and RF methods require larger dataset for training [24] and did not perform as well as PLSR. Support vector machine provided reduced performance compared to PLSR, and would also be expected to improve in performance relative to PLSR with a larger dataset. The various multiple linear regression models provided validation R^2^ of around 0.6, although the performance of these models would likely decrease on an independent dataset as they less effectively deal with multi-collinear data compared to PLSR based methods.

**Table 2:** Model performance of HSI models for nitrogen (PS + BL) (%) (ryegrass blades (BL) + pseudostem (PS))

HSI, Nitrogen (BL + PS)	R^2^ calibration (N = 127)	RMSE calibration (%)	R^2^ validation (N = 64)	RMSE validation (%)
PLSR (AW)	0.86	0.25	0.70	0.35
PLSR (AW0.99)	0.78	0.31	0.71	0.34
PLSR (CARS)	0.36	0.53	0.62	0.39
PLSR (VIP)	0.63	0.40	0.63	0.38
GPR	0.45	0.49	0.30	0.53
SVM	0.69	0.37	0.60	0.40
RF	0.67	0.38	0.14	0.58
MLR	0.77	0.32	0.67	0.36
SMLR	0.76	0.32	0.62	0.39
LASSO	0.73	0.34	0.65	0.37
RMLR	0.70	0.37	0.51	0.44

Calibration based on 66% data, with validation performed on the remaining 33% of data. Partial Least Squares Regression (PLSR) with latent variable selection based on the Adjusted Wold’s R criterion with thresholds on unity (AW) and 0.99 (AW0.99), Partial Least Squares Regression (PLSR) with wavelength selection according to Competitive Reweighted Adaptive Sampling (CARS) and Variable Importance Projections (VIP), Gaussian Process Regression (GPR), Support Vector Machine (SVM), Random Forest Regression (RF), Multiple Linear Regression (MLR), Stepwise Multiple Regression (SMLR), lasso regularization for linear regression (LASSO) and Robust Multiple Regression (RMLR)

We found that wavelengths in the range 900–1700 nm provided a much better prediction of total sugars than wavelengths in the 550–900 nm wavelength range with a calibration R^2^ of 0.53 (900–1700 nm) compared to 0.18 (550–900 nm) (Table 3). Conversely, we found that wavelengths in the 550–900 nm and the 900–1700 nm ranges each provided a similar prediction of nitrogen % with a calibration R^2^ of 0.62 (900–1700 nm) compared to 0.71 (550–900 nm) (Table 3). This highlights that the relative importance of wavelength ranges for the prediction of nitrogen and total sugars is dependent on the attribute. However, total sugars and nitrogen models based on the full wavelength range 550–1700 nm were superior to the respective individual VNIR (550–900 nm) and extended NIR (900–1700 nm) models, which highlights the value of obtaining spectral information over a broad range of wavelengths for accurate prediction of these attributes. Key wavelengths for the prediction of total sugars (based on PLSR(VIP)) are 548–573, 642–750, 1332–1460, 1509–1519, 1558–1627, and 1676–1696 nm. Key wavelengths for the prediction of nitrogen (based on PLSR(VIP)) are 548–582, 637–755, 888–893, 932–937, 1351–1415, and 1514–1696 nm.

**Table 3:** Model performance over different wavelength ranges

Variable	HSI data	Wavelength range	# LV PLSR	R^2^ calibration	RMSE calibration	R^2^ validation	RMSE validation	Validation intercept	Validation slope	Validation prediction bias
Sugars (mg/g)	BL + PS	550–1700 nm	12	0.70	28.9	0.58	34.4	10.1 ± 9.6 mg/g	0.93 ± 0.10	− 3.8 mg/g
Sugars (mg/g)	BL + PS	550–900 nm	5	0.18	47.6	0.20	47.7	− 15.3 ± 27.6 mg/g	1.20 ± 0.30	− 2.54 mg/g
Sugars (mg/g)	BL + PS	900–1700 nm	8	0.53	36.0	0.43	40.1	18.8 ± 11.6 mg/g	0.85 ± 0.12	− 4.9 mg/g
Nitrogen (%)	BL + PS	550–1700 nm	20	0.86	0.25	0.70	0.35	0.69 ± 0.22%	0.80 ± 0.067	− 0.05%
Nitrogen (%)	BL + PS	550–900 nm	13	0.71	0.36	0.41	0.48	1.19 ± 0.31%	0.64 ± 0.10	− 0.06%
Nitrogen (%)	BL + PS	900–1700 nm	7	0.62	0.41	0.44	0.47	0.64 ± 0.37%	0.79 ± 0.11	0.04%

HSI data, mean reflectance spectra analysed by PLSR model (Blades (BL) + pseudostems (PS) using Standard Normal Variate (SNV) preprocessing. Models are based on 550–1700 nm (original model based on full spectral range) and restricted wavelength ranges of 550–900 nm and 900–1700 nm

**Fig. 4:**
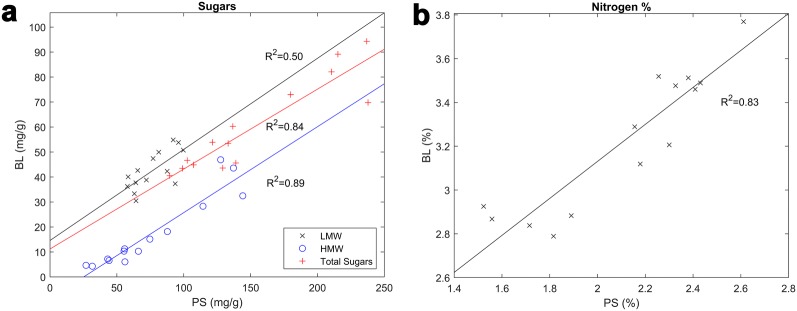
**a** Relationships between the measured sugar concentrations in the pseudostems (PS) and blades (BL) of the 15 ryegrass plants with wet chemistry data. There are positive relationships for measured high molecular weight sugar (HMW) (R^2^ = 0.89, RMSE = 4.9 mg/g, slope = 0.34 ± 0.03), low molecular weight sugar (LMW) (R^2^ = 0.50, RMSE = 5.6 mg/g, slope = 0.36 ± 0.10), and total (R^2^ = 0.84, RMSE = 7.7 mg/g, slope = 0.32 ± 0.04). Total sugar concentration is 100–250 mg/g at the base of the sward (PS) and 40–100 mg/g at the top of the sward (BL). **b** Relationship between the measured total nitrogen % in the pseudostems (PS) and blades (BL) of 15 ryegrass plants with wet chemistry (R^2^ = 0.83, RMSE = 0.14%, slope = 0.84 ± 0.11). Nitrogen concentration is 1.5–2.5% at the base of the sward (PS) and 2.5–4% at the top of the sward (BL)

There were differences in the nitrogen, total sugar, LMW and HMW sugar concentration between pseudostems and leaves in the 15 plants with wet chemistry measurements (Fig. 4). The pseudostems had a nitrogen concentration of 2.1 ± 0.35% whereas the blades had a nitrogen concentration of 3.2 ± 0.32% (difference of 1.1 ± 0.13% (*P* < 0.001)). Higher nitrogen concentrations in the blades are consistent with other studies that observed that the nitrogen concentration in leaves was ~ 50% greater than the stems of 99 day old perennial ryegrass plants [25] and mature ryegrass [26], although this is dependent on season and the time period after harvesting. In contrast, the pseudostems had a larger total sugar concentration of 153 ± 53 mg/g compared to leaves with a concentration of 60 ± 18 mg/g (difference of 93 ± 15 mg/g (*P* < 0.001)). This difference was associated with a difference (*P* < 0.001) in the HMW sugars between the pseudostems (75 ± 39 mg/g) and leaves (17 ± 14 mg/g) and a similar difference (*P* < 0.001) in the LMW sugars between the pseudostems (77 ± 15 mg/g) and leaves (43 ± 7.5 mg/g). LMW sugars are 2.5 times more concentrated in the leaves than the pseudostems compared to HMW sugars. Plants with high concentrations of nitrogen, total sugar, LMW or HMW sugar in the leaves also had corresponding high concentrations of nitrogen, total sugar, LMW or HMW sugar in the pseudostems (R^2^ = 0.50–0.89; *P* < 0.01).

**Fig. 5:**
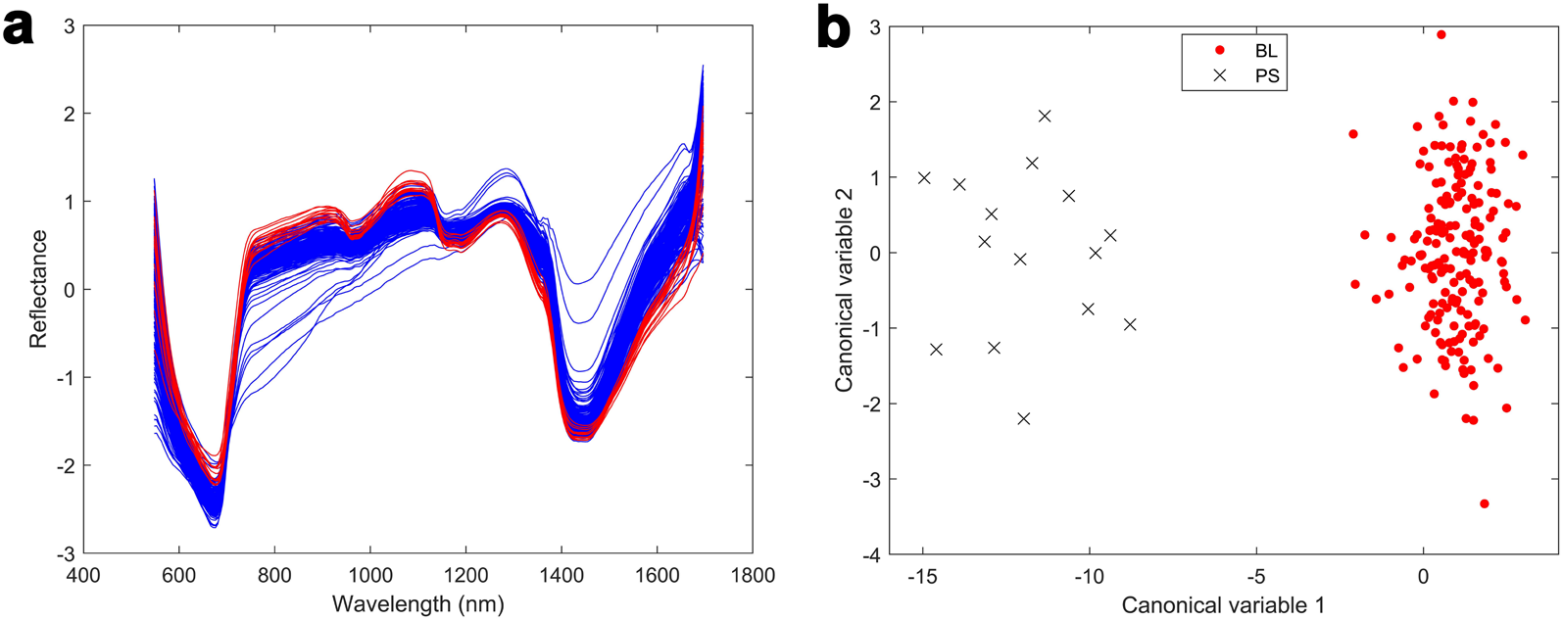
**a** Differences in the mean HSI spectra (SNV) between leaves (blue) and pseudostems (red). **b** Grouped scatter plot of the first two canonical variables obtained from a one-way multivariate analysis of variance of the PS + BL mean HSI spectra (n = 200). The first canonical variable provides a clear separation between ryegrass genotypes with blades (black crosses) and pseudostem (red circles)

There are significant differences between the mean spectra of PS and BL plants (Fig. 5a). Pseudostems have a characteristic high reflectance at 800–1100 nm (reflectance of 1.1 for PS compared to 0.7 for BL at 1100 nm) and a characteristic low reflectance at 1200–1500 nm respectively (reflectance of -1.2 for PS compared to -0.7 for BL at 1500 nm). The spectra were used to classify the genotypes into PS and BL groups (Fig. 5b). This highlights that the spectral profiles of the pseudostems and leaves of ryegrass plants are different.

**Fig. 6:**
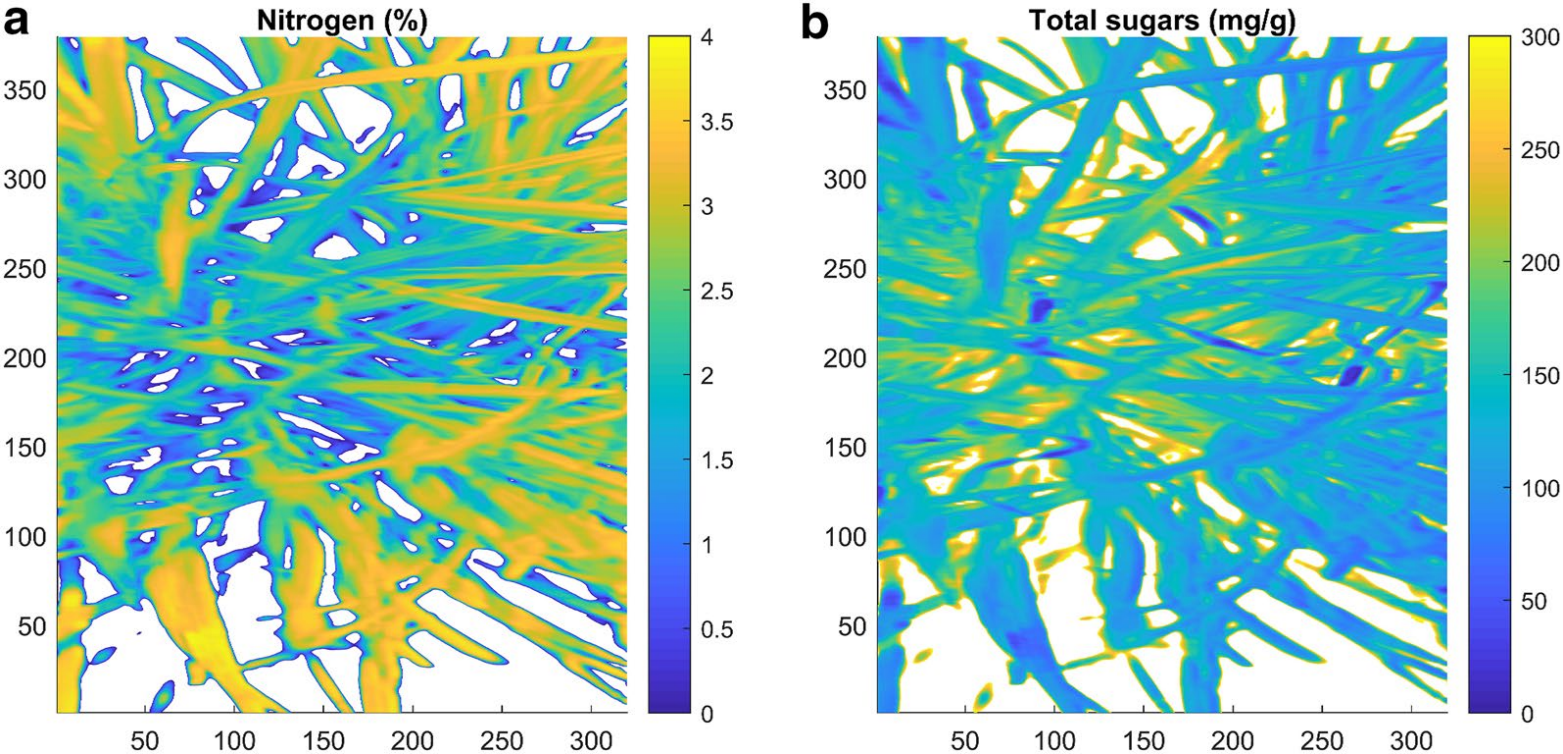
**a** The HSI predicted distribution in % nitrogen in the same plant shown in Fig. 1a. White denotes the background and regions with very low reflectance. Nitrogen concentration is 1–2.5% at the base of the sward (PS) and 2.5–4% at the top of the sward (BL). **b** The HSI predicted distribution in the total concentration of sugars (mg/g) in the same plant shown in Fig. 1a. White denotes the background and regions with very low reflectance. Total sugar concentration is 100–250 mg/g at the base of the sward (PS) and 40–100 mg/g at the top of the sward (BL)

The HSI predicted distribution in the concentration of nitrogen and total sugars prior to the first cut are shown in Fig. 6a, b respectively for the image in Fig. 1a. These predictions are independent of the lighting gradients inherent with the experimental setup illustrated in Fig. 1a. Nitrogen concentration was predicted to be 1–2.5% at the base of the plant (PS) and 2.5–4% at the top of the plant (BL). The total sugar concentration was 100–250 mg/g at the base of the plant (PS) but only 40–100 mg/g at the top of the plant (BL). These predictions for the spatial distribution of nitrogen and total sugars within the plant are consistent with the significant differences in the wet chemistry measurements of nitrogen and total sugars between the blades and pseudostems (Fig. 4a, b). This highlights the ability of hyperspectral systems to predict not only between plant differences in attributes, but also the variation in these attributes within a single plant.

## Discussion

The models developed in this study provide a rapid screening tool for selection of the best genotypes without the need for ongoing wet chemistry measurements. However, our calibration dataset of 120 plants is not large enough to cover a broader range of these traits. Therefore, these prediction models are expected to improve for calibration datasets with a larger number of samples such as the 1000–4000 laboratory analyzed samples in larger studies [9, 27]. Despite this, our models provide accurate, repeatable and rapid prediction of ryegrass quality traits under field conditions. In addition, our predictions of plant chemistry potentially provides a method to classify different plant species that have different chemical composition [28–30] (e.g. clover and/or weeds) for mixed sward-based applications such as high/low nitrogen species content and/or nitrogen fertilizer delivery [13, 14].

Hyperspectral data can be used to obtain information on the quality of forage on both spatial and temporal scales [8, 10]. This extra information can be used to parameterize mathematical models of forage growth and their response to environmental and management variables (e.g. climate and grazing) [31]. Hyperspectral data also provide information for testing the underlying assumptions of these mathematical models and revision and improvement of the models accordingly. The amalgamation of hyperspectral data with plant modelling will also allow for in-field assessment of plants and their diurnal and seasonal nutrient flows. These are traits and interactions that are difficult and time-consuming to objectively measure otherwise.

The cross-validation error (RMSE calibration) for NDF (RMSE = 2.53%), soluble sugars (2.89%), DOMD (1.36%) obtained by HSI (550–1700 nm, raw pasture) in this study are smaller or comparable to those obtained by NIRS by Corson et al. [4], for NDF (calibration RMSE = 2.79%), soluble sugars (calibration RMSE = 1.38%), DOMD (calibration RMSE = 3.37%) (1100–2500 nm, 60 °C dried and ground pasture samples). However, the NIRS model developed by Corson et al. [4], was calibrated over a wider range of NDF (17.8–78.0%), soluble sugar (1–25%) and DOMD (55–85%) values. Furthermore, the cross-validation error (RMSE calibration) for ADF (RMSE = 1.39%) and Ash (RMSE = 0.66%) are smaller or comparable to those obtained by NIRS by Pullanagari et al. [32], for ADF (RMSE = 2.13%) and Ash (RMSE = 0.74%) (350–1500 nm for field measured samples over a sample area of 0.25 m^2^ using the ASD FieldSpec^®^ Pro FR spectroradiometer), although our samples are not calibrated over as wide an attribute range.

Our current models were based on 550–1700 nm waveband range and are likely to be less informative than models calibrated over a broader range of wavelengths (350–2500 nm) [3, 7, 32]. We found that wavelengths in the extended NIR range 900–1700 nm provided a much better prediction of total sugars than wavelengths in the visible near-infrared 550–900 nm wavelength range. However, the two ranges provided a similar prediction of % nitrogen. This indicates that low cost visible near-infrared 400–1000 nm devices [24] are likely to provide satisfactory predictions of nitrogen but are likely to provide poorer prediction of the concentration of sugar in forage.

Differences in the nitrogen, total sugar, LMW and HMW sugar concentration between pseudostems and leaves of the same genotypes and even individual plants show the imbalanced distribution of these components in perennial ryegrass. The significantly higher nitrogen concentration in leaves than in the pseudostems was consistent with other studies [25]. The very high sugar concentration in pseudostems compared to the leaves was also consistent with previous studies using destructive methods, although this was dependent on the ryegrass cultivar, plant age and time of day [20, 26, 33]. We also used our model calibrations to predict the distributions in the concentrations of nitrogen and sugar within an individual plant at an individual pixel scale (sub leaf scale). Our predictions in the spatial distribution in nitrogen and total sugars within the plant were consistent with the measured differences in nitrogen and total sugars between stems and leaves in studies using destructive measures [20, 25, 26, 33]. This highlights the potential for hyperspectral imaging to predict the concentration and distribution of the components within an individual plant and the opportunity to conduct non-destructive and continuous experiments on the changes in the nutrient distribution within ryegrass plants.

The high positive correlation of nitrogen, total sugar, LMW or HMW sugar in the leaves with those in the pseudostems indicates that models calibrated to material from the upper zones of the plant will provide biased predictions of the average concentration of these components within the entire plant. The partitioning of sugar is consistent with previous studies of the distribution of sugars within ryegrass that found that the concentration of total sugar in the pseudostem was more than twice that in the leaf, although dependent on the cultivar, plant age and time of day [20, 26, 33]. However, the whole plant predictions of these traits will be correlated with the actual concentrations of these traits. The slope, intercept and error variance for this relationship, however, will depend on the ryegrass cultivar, plant age, time of day and plant structure. This highlights that bias and error can be introduced when extrapolating model predictions to regions of forage that have not been subject to hyperspectral calibration analysis. The identification of the differences in the spectral signatures of the pseudostems and leaves can be used to define the region of interest in an image for trait prediction (e.g. only in the leaves). It can also be used for spectral un-mixing procedures for forage imaged at greater distances from the ground [34].

Although the same approach and procedures can be used for other species of grasses, there will be species-specific challenges as the shape, shading and leaf overlap will vary across species. Furthermore, each species has its own chemical variation in different organs or sections of the plant. Therefore, species-specific models may be required for more accurate prediction of traits of interest.

## Conclusions

This study examines the utility of Hyperspectral Imaging (HSI) based methods for non-invasive assessment of the composition of ryegrass. The quality of forage is an important component of animal performance and environmental impact in pasture based production systems. Hyperspectral image data (550–1700 nm) were obtained from 185 individual ryegrass (*Lolium perenne*) plants that were also assessed for 13 forage quality attributes including nitrogen and sugar content. We used this data to develop models to predict these quality attributes of ryegrass and these provide for more accurate, repeatable and rapid prediction of ryegrass quality attributes under field conditions. We also examined ten different chemometric methods for predicting the forage attributes and established that partial least squares regression models performed favorably for the size of our calibration dataset. We also examined the relative importance of different wavelengths for the prediction of the different quality attributes and these can be used to make informed decisions about suitable sensors for field deployment of spectral systems. We also observed significant differences in the concentrations of nitrogen and sugars between the pseudostems and leaves of the plants and demonstrated the ability for hyperspectral systems to predict these differences within the plant. The use of hyperspectral systems will allow for more rapid genetic selection of desirable ryegrass attributes and provides an in-field tool to investigate the interactions between forage genetics, animal production and environmental outcomes in pastoral-based agricultural systems.

## Methods

### Plant material

Thirty-seven wild accessions and cultivars of perennial ryegrass (Lolium perenne L.) plants were clonally replicated (n = 5) and grown in individual pots in a fully-randomised complete block (n = 185 plants in total), outdoors at the AgResearch Grasslands campus in Palmerston North, New Zealand (40.3804°S, 175.6138°E). Plant seeds originated from different European countries, NZ and Australia. Clones were produced by dividing one plant into five 5-tiller plants and potted in prepared soil mix with slow release Osmocote. Plants were kept outside but under overhead irrigation so that the plants did not dry out during periods with no rain. Plants were 3 months old when harvested. For each plant, total leaf material was harvested, within 1 min after hyperspectral scanning, by cutting at 4 cm above the soil surface. Leaf material was immediately snap-frozen in liquid nitrogen. For a subset of three genotypes × 5 clonal replicates (n = 15 plants), pseudostems (PS) and upper leaf blades (BL) were scanned and harvested separately. All samples were held at − 80 °C prior to freeze-drying. Freeze-dried material was milled and subsequently analysed by wet chemistry. The ratio of leaf blade: pseudostem was also recorded for each plant.

### Wet chemistry

The low molecular weight (LMW), high molecular weight (HMW) and total sugars for each plant were measured via water soluble carbohydrate (WSC) analysis and expressed as mg/g (mean of 3 replicates). Ash (%), nitrogen (%), neutral detergent fibre (NDF; %), acid detergent fibre (ADF; %), DOMD (% DM), ME (MJ/kg DM), DM (%), Ca (mg/g) and OM (%) were also measured for each plant. This provided a data set of 185 samples for leaf blades or lamina and an additional 15 samples for pseudostems.

WSC were determined by liquid/gas chromatography–mass spectrometry based technique [35]. Dry matter and Ash/OM were determined by AOAC 930.15/925.10/942.05. For Ash determination the sample was ignited at 500 °C to burn off all organic material. The inorganic material not volatilized at this temperature is the ash. For dry matter determination the moisture of the sample was removed by volatilization caused by heating at 105 °C for 16 h. The amount of material left after the removal of the moisture was defined as the dry matter. Total Nitrogen was determined by Leco CN analyzer, AOAC 968.06 [36]. The sample was weighed into tin foil capsule, loaded into the furnace and combusted in a stream of oxygen. The products of combustion were then passed through a secondary furnace for further oxidation and particulate removal. The moisture free gases were then swept through a heated copper catalyst under helium flow to remove oxygen and convert NOx to N2, and the nitrogen content was determined with a thermal conductivity cell. The crude protein content of the sample was obtained by multiplying total nitrogen content by 6.25. Calcium was determined by preparation AOAC 968.08D followed by colourimetric analysis. The estimation of DOMD followed the method of Roughan and Holland [37]. NDF/ADF were determined by Ankom. Metabolizable Energy (ME) was determined by calculation.

### Hyperspectral imaging

The Hyperspectral Line Scanning Imaging System (Hyperspec^®^ Extended VNIR, Headwall Photonics, Fitchburg, MA, USA) with 235 wavebands captured between 550 and 1700 nm for each of 320 pixels in a line and 400 lines per image was used to capture spectra from the samples. Hyperspectral images were captured before and after harvesting the top half of the plant (BL and PS images respectively). A 50 mm lens with a focal length of 25 mm, pixel pitch of 30 µm and an f-stop of 2.8 were used for imaging all samples. A moving stage system was used at a speed of 11.1 mm/s and with an image write speed of 25 frames per second. The spatial resolution was calculated by the Hyperspec™ software and the HSI camera exposure setting was fixed at 28 ms. A single line beam halogen light source (30^o^ angle of incidence from vertical, 400 W) was used to illuminate samples and all 185 plants were scanned. Measurements were undertaken on the 22nd of March 2016 in a dark room at the AgResearch Grasslands site, Palmerston North, New Zealand. A total of 452 images were collected with either two or three images per plant from directly above depending on the quality of image capture and the general morphology of each plant. White and dark reference samples were collected for each image with a 200 mm × 50 mm Spectralon white reference tile and the lens cap on respectively. The reflectance (*R*) of each pixel/sample was calculated using these white (*W*) and dark (*D*) reference samples according to the equation

1 $$R = \frac{I - D}{W - D}$$

where the calibrated image reflectance is obtained from the raw image irradiance (*I*) and the dark and white reference images.

### Analysis

Approximately 128,000 spectra were collected per hyperspectral image, however, only a portion of these contain information about the sample of interest. An algorithm was developed to automatically segment the images into ryegrass regions of high spectral reflectance. This was defined by the region with R ≥ 0.3 (reflectance at 1080 nm, which was chosen as this wavelength is not much affected by water content), which was representative of the harvested region.

Multivariate analysis of the spectra was conducted using partial least squares regression (PLSR). Partial least squares regression is a widely accepted regression modelling approach that effectively deals with multi-collinear data [38]. Models tested were based on the mean spectrum. The mean spectrum were calculated from 29,228 ± 18,255 HSI pixels, which represents on average 23% of the total number of HSI pixels. Models were based on the average spectrum from the 2–3 replicate images captured per plant. In total, three plants with an average number of pixels less than 5000 were not used in the analysis. Reduced models were also evaluated based on the average of 1, 2 or 3 images captured per plant. Models for visual yield also included the number of pixels as a predictor. Models were developed using two methods: 1) only the blades material (BL) and 2) the blades and pseudostem material (BL + PS).

The predictive models were calibrated and cross-validated using two-thirds of the data set. The calibration data set was used to fit the chemometric models that allow forage composition to be predicted from spectral data obtained from HSI. The remaining one-third of the data set was used for model validation.

Standard normal variate (SNV) pre-processing methods were applied to the mean spectral data [39]. The number of latent variables for PLSR was identified using tenfold cross-validation (CV) with 100 Monte Carlo replicates applied to the calibration data set. Models were built using varying numbers of latent variables (LV) from 1 to 50 and applied to the validation set to generate performance values of R^2^ and root mean squared error (RMSE) [40]. Based on the regression Y_measured_ = a + b × Y_predicted_, the validation slope (b), intercept (a) and bias (expected predicted-observed) were also calculated. The optimal number of latent variables is determined by selecting the calibration model with the smallest RMSE (Adjusted Wold’s (AW) R criterion with threshold of unity and 0.99; denoted AW and AW0.99) and results are reported for the AW criteria unless otherwise specified.

Models were also obtained based on Partial Least Squares Regression (PLSR) with wavelength selection according to Competitive Reweighted Adaptive Sampling (CARS) and Variable Importance Projections (VIP), Gaussian Process Regression (GPR), Support Vector Machine (SVM), Random Forest Regression (RF), Multiple Linear Regression (MLR), Stepwise Multiple Regression (SMLR), lasso regularization for linear regression (LASSO) and Robust Multiple Regression (RMLR) [27].

Models were explored using the data over the wavelength ranges 550–900 nm and 900–1700 nm separately to investigate the likely predictive power of different wavelength ranges from visible to extended NIR available in hyperspectral cameras at AgResearch.

Based on the PLSR(VIP) analysis we determined that 558 nm and 740 nm are key wavelengths for both nitrogen and sugars and 937 nm is a key wavelength for nitrogen estimation. The wavelengths 558 nm and 740 nm are also influenced by chlorophyll content. For this reason 558, 740, and 937 nm were selected as wavelengths to visualize the HSI images of forage (Fig. 1).

Multivariate linear regression was also used to investigate the effect of plant material (BL or PS) and plant genotype on the mean HSI spectra [41]. One-way multivariate analysis of variance was also used to compare the multivariate means in the mean HSI spectra data grouped by plant material type (PS, BL). The analysis was based on spectral data from every 2nd wavelength (117 bands). Scatter plots were used to visualize the group separation using the first two canonical variables.

## Data Availability

The datasets used and/or analysed during the current study are available from the corresponding author on reasonable request.
